# Intrafascial Supracervical Hysterectomy Without Colpotomy and Transuterine Mucosal Resection by Pelviscopy and Laparotomy

**DOI:** 10.1155/DTE.1.201

**Published:** 1995

**Authors:** Liselotte Mettler, Erick Alvarez-Rodas, Enrique Lehmann-Willenbrock, Jutta Lüttges, Kurt Semm

**Affiliations:** 1 Departments of Obstetrics and Gynecology Christian Albrechts University of Kiel Germany; 2 Department of Pathology Christian Albrechts University of Kiel Germany; 3 Michaelisstrabe 16 Kiel 24105 Germany

## Abstract

Between September 1991 and December 1993, 253 patients were operated on using the Classical Intrafascial SEMM (Serrated Edged Macro Morcellator) Hysterectomy (CISH) technique. One hundred fifty-two patients were assigned to pelviscopic CISH and 101 to laparotomic CISH. Uterine leiomyomas with menstrual disorders and pressure symptoms topped the list of indications with 61%. In all cases, initially transuterine mucosal resection and coring of the cervicouterine cylinder were carried out followed by the intrafascial supracervical dissection of the uterus. The size of the uterus played a decisive role in selecting the cases for CISH technique either by pelviscopy or laparotomy. The cervicouterine mucosal cylinders were cored using the Calibrated Uterine Resection Tool (CURT). Cervical thickness and diameters were measured preoperatively by transvaginal sonography for facilitating the use of a specific-sized CURT. After removal of this cylinder, hemostasis in the area was secured by coagulating with an endocoagulation device. The advantage of this technique is that the pelvic floor integrity remains intact, and because uterine arteries and ureters were not touched, the so called “complication zone” is thus avoided.

The histological findings are in agreement with the indications, the leiomyomas and leiomyomas with adenomyosis being the most frequent pathology. The histologic analysis showed that in all cases the squamocolumnar transformation zone was totally removed. There were 11 (4.4%) complications, promptly identified and treated without further problems.

The value of the Classical intrafascial supracervical hysterectomy without colpotomy including the resection of transformation zone speaks for itself, because there is less physical stress and recovery is quick. However, it has yet to prove its value as compared with other techniques for hysterectomy for specific indications.


*Diagnostic and Therapeutic Endoscopy*, 1995, Vol. 1, pp. 201–207
Reprints available directly from the publisher
Photocopying permitted by license only

© 1995 Harwood Academic Publishers GmbH
Printed in Singapore

### Intrafascial Supracervical Hysterectomy without Colpotomy and Transuterine Mucosal Resection by Pelviscopy and Laparotomy

LISELOTTE METTLER,[1] ERICK ALVAREZ-RODAS,[1] ENRIQUE LEHMANN-WILLENBROCK,[1] JUTTA LÜTTGES,[2] and KURT SEMM[1]

*From the [1]Departments of Obstetrics and Gynecology, and the [2]Department of Pathology, Christian Albrechts University of Kiel, Germany.*

*(Received August 15, 1994; in final form October 31, 1994)*

Between September 1991 and December 1993, 253 patients were operated on using the Classical Intrafascial SEMM (Serrated Edged Macro Morcellator) Hysterectomy (CISH) technique. One hundred fifty-two patients were assigned to pelviscopic CISH and 101 to laparotomic CISH. Uterine leiomyomas with menstrual disorders and pressure symptoms topped the list of indications with 61%. In all cases, initially transuterine mucosal resection and coring of the cervicouterine cylinder were carried out followed by the intrafascial supracervical dissection of the uterus. The size of the uterus played a decisive role in selecting the cases for CISH technique either by pelviscopy or laparotomy. The cervicouterine mucosal cylinders were cored using the Calibrated Uterine Resection Tool (CURT). Cervical thickness and diameters were measured preoperatively by transvaginal sonography for facilitating the use of a specific-sized CURT. After removal of this cylinder, hemostasis in the area was secured by coagulating with an endocoagulation device. The advantage of this technique is that the pelvic floor integrity remains intact, and because uterine arteries and ureters were not touched, the so called "complication zone" is thus avoided.

The histological findings are in agreement with the indications, the leiomyomas and leiomyomas with adenomyosis being the most frequent pathology. The histologic analysis showed that in all cases the squamocolumnar transformation zone was totally removed. There were 11 (4.4%) complications, promptly identified and treated without further problems.

The value of the Classical intrafascial supracervical hysterectomy without colpotomy including the resection of transformation zone speaks for itself, because there is less physical stress and recovery is quick. However, it has yet to prove its value as compared with other techniques for hysterectomy for specific indications.

**KEY WORDS:** laparoscopy, minimally invasive surgery, hysterectomy, laparoscopic hysterectomy, laparoscopic-assisted vaginal hysterectomy

### INTRODUCTION

A new technique for pelviscopic and laparotomic hysterectomy, the Classical Intrafascial SEMM Hysterectomy (CISH) has been recently described (1). This technique pretends to combine the advantages of the traditional supracervical hysterectomy, including a shorter operative time and the preservation of the cardinal ligaments and pericervical tissue, with the prevention against cervical carcinoma (2).

Address for correspondence: L. Mettler, M.D., Michaelisstrabe 16, 24105 Kiel, Germany

Whereas first carefully described abdominal supracervical hysterectomy was performed by Wilhelm Alexander Freud in 1878 (3,4), it was the leading technique for over 80 years. The shift to total hysterectomy occurred because of the danger of cervical stump cancer (5). In 1936 Tervilä (6), described the danger of cervical cancer to be 0.3% to 1.9% following the supracervical hysterectomy. In 1991 followed the intrafascial supracervical hysterectomy without colpotomy (1).

The advantages of this CISH technique (by both pelviscopy and laparotomy) are preservation of the integrity of the pelvic floor (nerval and vascular side); continuation of the normal sexual life for both partners; and protection

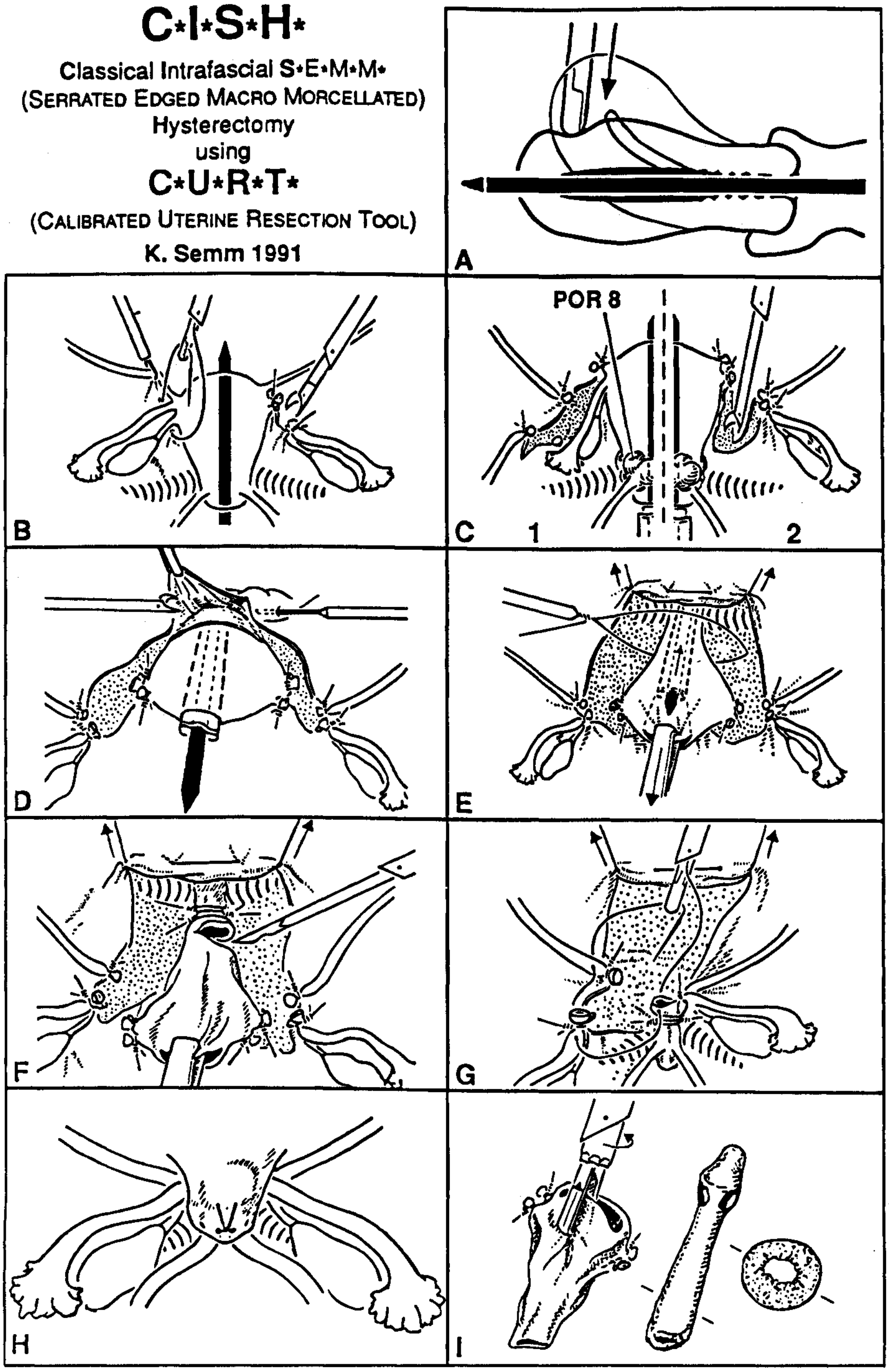

**Figure 1** Steps of the Classic Intrafascial SEMM (Serrated Edged Macro-Morcellator) Hysterectomy—CISH
*A*, Introduction of the perforation rod in the uterine cavity.
*B*, Pelviscopic adnexal dissection.
*C*, Dissection of the broad ligament.
*D*, Bladder dissection, coring out of the cervicouterine mucosa.
*E*, Roeder's loop is closed around the cervix.
*F*, Uterine resection above the loops.
*G*, Stump suspension to the round ligaments.
*H*, Stump peritonialisation.
*I*, Uterus morcellation and extraction. View of the cylinder specimen.

against carcinoma of the cervix by coring out the complete transformation zone.

This paper summarizes our experience with CISH technique and evaluates this procedure as performed in our Department of Obstetrics and Gynecology in Kiel via pelviscopy and laparotomy. The indication for the CISH technique is clearly given for benign alterations of the uterus.

### MATERIAL AND METHODS

Between September 1991 and December 1993, CISH procedures were performed on 253 women: pelviscopic CISH on 152 and laparotomic CISH on 101. The patients were assigned depending on the size of the uterus and the skill of the surgeon. The endoscopic technique was limited to 12-week pregnancy-sized uterus for technical ease (7). Forty-two patients received GnRH analogues preoperatively to reduce the size of the myomas, with different preparations and doses' schemes. The operations were performed by a group of 10 surgeons. The haemoglobin count was controlled systematically between 24 and 48 hours after hysterectomy. Blood loss was estimated by the surplus of aspirated fluid within the irrigation suction process.

A complete description of the CISH technique is given by Semm (1). Briefly the procedure is as follows—the diameter of the cervix is estimated preoperatively by bimanual palpation and/or vaginal ultrasound.

### Pelviscopic CISH Technique

The procedure is carried out in methodical vaginal and pelviscopic steps.

#### *1st Vaginal Step*

The cervix is grasped at 3 o'clock and 9 o'clock positions with two tenaculae. POR-8® is injected into the cervix. The solution contains 0.05 I.U. of vasopressin per mL. Dilatation from Hegar 2 to 6 is carried out before introducing the CURT guide rod through the fundus of uterus so as to manipulate the uterus as desired. Tenaculae are fixed with the guide rod by the fixation screws for easy manipulation.

#### *1st Pelviscopic Step*

After a routine panoramic inspection of the entire peritoneal cavity, careful inspection of the minor pelvis is carried out by pushing the bowels towards the diaphragm. The patient is now brought into the Trendelenburg-position. Adnexal dissection is carried out bilaterally with sutures and ligatures or staplers until the level of the cardinal ligaments. Acquadissection of bladder comes next. A Roeder loop now is placed loosely around the cervix, avoiding contact with the uterine vessels and the ureters.

#### *2nd Vaginal Step*

The cervicouterine mucosa is resected with the CURT by careful coring. The size of CURT is selected according to the preoperative measurement of the cervical diameter by ultrasound. The remaining part of the cervix is endocoagulated at 100° to 120° C by means of thermocoagulation.

#### *2nd Pelviscopic Step*

The Roeder loop placed around the cervix before the 2nd vaginal step is now tightened to prevent the loss of gas. The cervix is further secured with two more Roeder loops. The uterus is resected with the hook scissors above the level of the loops. After disinfecting the cervical stump with an antiseptic swab, it is suspended with the round ligaments to maintain the pelvic floor. The visceral peritoneum is closed over the cervical stump, and the uterus is morcellated and extracted.

### LAPAROTOMY CISH TECHNIQUE

After performing a subtotal hysterectomy using the classical method, the perforation rod is introduced through the cervical stump. The cervical tissue is cored out and endocoagulation is applied.

### RESULTS

The analysis of the CISH cases showed a mean age of 47.6 years (Table 1). No difference in age or mean parity (1.3) was found between the pelviscopic and laparotomic groups. Leiomyomas with diverse clinical presentations were the main indication to CISH and were diagnosed in 80% of the laparotomic and 71% of the pelviscopic cases. Other indications were abnormal uterine bleeding that persisted despite medical or conservative surgical treatment, chronic pelvic pain, and endometriosis. The uterocervical length ultrasonographically measured ranged between 6 to 13.5 cm, midlength 8.9 cm for the pelviscopic CISH cases, and the midlength of the laparotomic CISH patients was 15.3 (range 7.5 to 28 cm). The cervical thickness found was between 1.6 and 3.5 cm in 244 (97.2%) of the hysterectomized patients. (Table 1)

The size of the uterus played an important role in selecting the patients for pelviscopic or laparotomic CISH, as shown by the weight difference between the pelviscopic

Table 1 Pre Operative Data of 253 CISH Cases

| | *Laparotomy* | *Pelviscopy* |
|---|---|---|
| Mean (range) age (yrs) | 48.3 (33–65) | 47 (32–68) |
| Mean (range) Parity (delivery) | 1.3 (0–5) | 1.4 (0–5) |
| **Indications** | | |
| Leiomyomas with menstrual abnormality | 46 (45%) | 73 (48%) |
| Leiomyomas with pressure symptoms | 35 (35%) | 35 (23%) |
| Therapy resistant dysfunctional uterine bleeding | 8 (8%) | 34 (22%) |
| Chronic pelvic pain | 6 (6%) | 7 (5%) |
| Endometriosis | 6 (6%) | 3 (2%) |
| **Uterocervical length ultrasonographically measure** | | |
| <8 cm | 22 (21.8%) | 62 (40.8%) |
| >8 cm | 79 (78.2%) | 90 (59.2%) |
| **Cervical thickness ultrasonographically measured** | | |
| >1.5 cm | 3 (3%) | 6 (4%) |
| 1.6–2.5 cm | 33 (32.7%) | 47 (31%) |
| 2.6–3.5 cm | 65 (64.3%) | 99 (65%) |

Note.—CISH indicates classical intrafascial SEMM (serrated edged macro morcellator) hysterectomy.

and laparotomic specimens (mean 162 vs 372 g) (Table 2). Concomitant laparoscopic adhesiolysis was performed in 26 cases of CISH by laparotomy and 48 patients by pelviscopic CISH.

The average operative time for CISH by laparotomy was 125.5 (range, 60 to 190 minutes), and for CISH by pelviscopy was 152.2 (range, 60 to 220 minutes).

Estimated blood loss was minimum, with an average of 331.6 (range, 100 to 575 mL) for CISH by laparotomy and 315.3 (range, 50 to 600 mL) for CISH by pelviscopy. There was no significant difference in comparison with the preoperative blood count. Clinically, preoperative suppressive therapy appeared to reduce operative blood loss and time and specimen size. However, data analysis did not show a statistically significant difference. The average length of hospitalization was 4 to 7 days. The hospitals stay after a vaginal or abdominal hysterectomy in Germany is more than 10 days. Initially, hospitalization

Table 2 Transoperative and Postoperative Data of 251 CISH Cases

| | *Laparotomy* | *Pelviscopy* |
|---|---|---|
| **Diameter of CURT used** | | |
| 10 mm | 5 (5%) | 10 (7%) |
| 15 mm | 57 (56%) | 98 (64%) |
| 20 mm | 39 (39%) | 44 (29%) |
| **Weight chart of uteri** | | |
| Mean range (g) | 372 (50–2060) | 162.4 (45–480) |
| 0–100 | 9 (9%) | 58 (38%) |
| 101–200 | 31 (31%) | 71 (47%) |
| 201–400 | 35 (35%) | 20 (13%) |
| >400 | 26 (25%) | 3 (2%) |
| **Operative Time** | | |
| Mean (range) min | 125.5 (60–190) | 152.2 (60–220) |
| 60–90 | 24 (23.7%) | 17 (11%) |
| 91–120 | 25 (24.7%) | 30 (20%) |
| 121–180 | 36 (35.6%) | 62 (41%) |
| >180 | 16 (15.8%) | 43 (28%) |
| **Blood loss** | | |
| Mean (range) EBL (ml) | 331.6 (100–575) | 315.3 (50–600) |
| 0–250 ml | 23 (22.7%) | 40 (26.3%) |
| 251–500 ml | 48 (47.5%) | 66 (43.4%) |
| >500 ml | 30 (29.7%) | 46 (30.3%) |
| **Hospital stay** | | |
| Mean (range) days | 7.1 (3–10) | 7.1 (3–10) |
| <4 days | 6 (5.9%) | 10 (6.5%) |
| 4–7 days | 40 (39.6%) | 85 (56%) |
| 8–10 days | 55 (54.5%) | 57 (37.5%) |
| **Complications** | | |
| None | 100 (99%) | 142 (93.4%) |
| Hematoma/Retention cyst | 1 (1%) | 6 (4%) |
| Vaginal bleeding | — | 4 (2.6%) |

Note.—CISH indicates classical intrafascial SEMM (serrated edged macro morcellator) hysterectomy.

was longer mainly for evaluation and observation purpose, but with experience the length of stay was reduced significantly.

Eleven patients presented complication (Table 2); this demonstrates an overall 4.4% rate of complications. Only one (1%) case by laparotomy and 6 patients (4%) by pelviscopy had infected hematoma and a retention cyst, which required an antibiotic therapy. The remaining complications consisted of four (2.6%) cases of vaginal bleeding, which were necessary to stop by thermocoagulation.

Glandular tissue at the endocervical level was found at the edge of the cylinder in only 8% of the cases (11% of the laparotomic and 5% of the pelviscopic CISH), and there was no correlation with the diameter of the coring device used (Table 3). This may be the reason for us having selected to small coring devices.

The squamocolumnar transformation zone was totally removed by CURT in 100% of the cases. Cervical dysplasia was found in 15 (11.5%) histologic specimens and was not preoperatively diagnosed, although all the patients had a cervical smear within 1 year before surgery. On reviewing the cytological reports, we found that all the patients with dysplasia had a PAP I or PAP II cervical smear. The cervical dysplasia was limited to the cylinder specimen in all of the cases.

Eight (6.6%) patients had endometrial hyperplasia, and all of them were symptomatic (abnormal uterine bleeding). The diagnosis of hyperplasia was established by dilation and curettage in all eight cases and was not the main indication for CISH.

A histologic diagnosis of leiomyosarcoma was made in a 52-year-old patient. The clinical presentation consisted of menometrorrhagia of 6 months of evolution. The ultrasound examination showed a 15 × 18 × 16 cm enlarged uterus, and the dilation and curettage showed a normal endometrium. A laparotomic CISH was performed, and an intramural disseminated leimyosarcoma with linfo and hemangiomatose degeneration without endometrial invasion was found on histology. Postoperative radiotherapy was administered and 6 months after surgery the patient was totally recovered and asymptomatic

### DISCUSSION

Our 253 cases of CISH hysterectomy show that it can be applied without any major complication by an experienced surgeon. Because of carefully performed operations, we were able to avoid any complications intraoperatively. Although we had postoperative complications in forms of hematomas caused by slippage of ligature and resultant bleeding, vaginal bleeding caused by improper hemostasis in the remaining cervical stump. In our first 40 cases, no hemostatic endocoagulation was performed after the cervicouterine mucosa resection by CURT. We think that our careful approach would allow us to reduce the complications as low as mentioned above.

We think that our techniques using suture ligatures or staplers for hemostasis were quite satisfactory, because we had no intraoperative hemorrhagic accidents and only four cases of hematomas identified early. Mage et al. (8) and Nezhat et al. (9) in their technique for laparoscopically assisted vaginal hysterectomy (LAVH) also have successfully used staplers. Already in 1984 Semm (4) pub-

**Table 3** Histological Findings of 253 CISH Cases

| *Histological findings of uteri* | *Laparotomy* | *Pelviscopy* |
|---|---|---|
| Leiomyoma | 60 (59%) | 81 (53.3%) |
| Leiomyoma with adenomyosis | 24 (24%) | 21 (14%) |
| Adenomyosis | 9 (9%) | 40 (26%) |
| Adenomatous Hyperplasia | 3 (3%) | 5 (3.3%) |
| No remarkable findings | 4 (4%) | 5 (3.3%) |
| Sarcoma | 1 (1%) | — |
| **Histological findings of cylinder specimens** | | |
| Normal | 83 (82%) | 133 (87.5%) |
| Chronic cervicitis | 13 (13%) | 9 (6%) |
| Dysplasia | 5 (5%) | 10 (6.5%) |
| **Glandular findings on the edge of the cylinder specimen** | | |
| Free from glands | 90 (89%) | 145 (95%) |
| Glands reaching the border | 11 (11%) | 7 (5%) |
| Transformation zone | 101 (100%) | 152 (100%) |
| **Diameter of CURT used: in the cases with glands reaching the edge** | | |
| 10 mm | — | — |
| 15 mm | 8 (44.4%) | 1 (5.5%) |
| 20 mm | 3 (16.6%) | 6 (33.3%) |
| **Total** | **11 (61.1%)** | **7 (38.8%)** |

Note.—CISH indicates classical intrafascial SEMM (serrated edged macro morcellator) hysterectomy.

lished the first laparoscopic-assisted vaginal hysterectomy. In 1990 Reich[10] published his first endoscopic total hysterectomy; however, removal of the endoscopically resected uterus was via colpotomy (9,11).

The value of CISH compared with the vaginal hysterectomy still will have to be demonstrated. Several of the potential advantages of pelviscopy over the conventional abdominal approach, such as reduced physical stress, hospital stay, and economic costs are well-known advantages of vaginal surgery (12,13). Actually a comparison of the two techniques is difficult, because the vaginal technique has been improved constantly over 30 years (14). The fact that 70% of hysterectomies in the United States are performed by laparotomy (15,16) together with the tremendous recent progress in pelviscopic surgery, argues in favor of the pelviscopic approach. The findings of 15 patients (5.9%) with cervical dysplasia in our series underlines the great importance of an exhaustive preoperative diagnosis to exclude the possibility of cervical neoplasia; however, the fact that all patients underwent surgery without a diagnosis of dysplasia despite an appropriate routine screening for cervical carcinoma (ACOG, 1993) remarks the limitation of the cervical smear, and it is in accordance with the described false-negative rates of 20% to 30%. This series shows clearly that with the CISH technique the transformation zone is totally removed in 100% of the cases, reducing the possibility of a cervical stump neoplasia to a minimum. The total amount of functional cervical tissue was resected in 92% of the cases. Six months after surgery, the cases with glands reaching the edge of the cored out specimen underwent screening. Cervical pathology was not diagnosed in any of the cases. We believe that estimating with transvaginal ultrasonography the diameter of the cervix on a routine basis, it is possible to select the proper CURT diameter and achieve a 100% removal of the cervical glandular tissue. The leiomyosarcoma is an infrequent tumor that represents approximately 2% of the uterine malignant neoplasias.[18] Its clinical feature is usually that of an enlarging mass. Unless extrauterine disease occurs, it may be impossible to distinguish a sarcoma from a leiomyoma (5), as was the case with the patient presented in this series.

The CISH technique was conceived in an effort to combine all the advantages of the subtotal hysterectomy, regarding costs, time, risks, and possible complications, with the prevention of cervical carcinoma, through the transvaginal cylindrical coring out of the cervical tissue using the CURT and the pelviscopic CISH technique only over 4 years.

The total hysterectomy has several disadvantages inherent to its more radical character, such as longer operating time, greater amount of bleeding, and greater to adjacent organs specially ureters. Furthermore, the resection of the cervical tissue by the traditional method implies the destruction of the vascular and neuronal pericervical network, with a consequent impact in the sexual life of the posthysterectomized women; whereas there is no statistically significant change on the libido, their ability to achieve an orgasm is clearly impaired (6), and different authors state that the cervix should not be removed without proper indication.

Our technique of avoiding the dissection in the field of uterine vessels and the ureter has proved itself in the form that we have no vascular or urinary complications either intraoperatively or postoperatively. Overall, we sincerely feel that CISH is a technique of today that has its firm footings, but needs long-term follow ups.

### REFERENCES

1. Semm K. Hysterectomy per laparotomiam oder pelviscopiam ohne Kolpotomie, ein neuer Weg durch CASH Geburtshilf u. Frauenheilk 1991;51:996–1003.
2. Kilkku P, Grönroos M, Hirvonen T, et al. Supravaginal uterine amputation vs. hysterectomy. Effects on libido and orgasm. Acta Obstet Gynecol Scand 1983;62:147–152.
3. Freund WA. Bemerkungen zu meiner Methode der Uterus Exstirpatio Zent bl Gynäkol, 1878;2:497–500.
4. Freund WA. Method of complete removal of the uterus Am J Obstet, 1879;XII 200.
5. Semm K, Operationslehre für endoskopische Abdominal-Chirugie-Operative Pelviscopie. Schattauer Verlag Stuttgart, New York, 1984; Translation English Year book medical Publishers Inc. Chicago, London 1987.
6. Tervilä I. Carcinoma of the cervical stump. Acta Obstet Gynecol Scand 1963;42:200–206.
7. Mettler L, Semm K. Intrafascial supracervical hysterectomy without colpotomy and transuterine mucosal resection by pelviscopy or laparotomy: our first 100 cases. Presented at the third biennial meeting of the International Society of Gynecologic Endoscopy, Washington, DC, 1993;6:23–26.
8. Mage G, Canis M, Chapron Ch, Wattiez A, Pouly JL, Bruhat MA. Laparoscopic hysterectomy. J Gynecol Obst Biol Reprod 1990;19:573–576.
9. Nezhat C, Nezhat F, Silfen SL. Laparoscopic hysterectomy and bilateral salpingo oophorectomy using multifire GIA surgical stapler. J Gynecol Surg 1990;6:287–288.
10. Reich H, De Caprio J, Mc Glynn F. Laparoscopic hysterectomy. J Gynecol Surg 1989;5:213–216.
11. Reich H. Laparoscopic hysterectomy. J Gynecol Surg 1989;5:2.
12. Dicker RC, Greenspan JR, Strauss LT. Complications of abdominal and vaginal hysterectomy among women of reproductive age in the United States: the collaborative review of sterilization. Am J Obstet Gynecol 1982;144:841–848.
13. Reiner LJ. Early discharge after vaginal hysterectomy. Obstet Gynecol 1982;71:416–418.
14. Backman GA. Hysterectomy: a critical review. J Reprod Med 1990;35:839–862.
15. Dicker RC, Scally MJ, Greenspan JR. et al. Hysterectomy among women of reproductive age: trends in the United States 1970–1978. JAMA 1982;284:323–327.

16. Wingo PA, Huezo CM, Rubin GL, Ory HW, Peterson HB. The mortality risk associated with hysterectomy. Am J Obstet Gynecol 1985;152:803–808.
17. ACOG Comite Opinion, Committee on Gynecologic Practice. No. 128. October 1993: Routine cancer screening. Int J Gynecol Obstet 1993;43:344–348.
18. Eberl M, Pfleiderer A, Teufel G. et al. Sarcoma of the uterus: morphological criteria and clinical course. Path Res Pract 1980;169:165–167.
19. Pelosi MA, Pelosi MA. Laparoscopic supracervical hysterectomy using a single umbilical puncture: minilaparoscopy. J Reprod Med 1992;37:774–784.
20. Hasson HM, Rotman C, Rana N et al. Experience with laparoscopic hysterectomy. J Am Assoc Gynecol Laparosc 1993;1:1–11.
21. Mettler L, Semm K, Lüttges J, Panandikar D. Pelviskopische intrafasciale Hysterectomie ohne Kolpotomie (CISH). Gynak Prax 1993;17:509–526.

